# Transition of the genital mollicutes from the second to the third trimester of pregnancy and its association with adverse pregnancy outcomes in GDM women: a prospective, single-center cohort study from China

**DOI:** 10.1186/s12884-024-06418-x

**Published:** 2024-04-03

**Authors:** Yan Xuan, Jun Zhao, Xiang Hong, Tao Yan, Yue Zhang, Xu Zhou, Junhui Zhang, Bei Wang

**Affiliations:** 1 Key Laboratory of Environmental Medicine and Engineering of Ministry of Education, Department of Epidemiology and Statistics, School of Public Health, Southeast University, No. 87 Dingjiaqiao Road, Gulou District, Nanjing, Jiangsu China; 2 National Research Institute for Family Planning, Beijing, China; 3 National Human Genetic Resources Center, Beijing, China; 4 Health Management Center, the First Affiliated Hospital of Chongqing Medical University, Chongqing, China

**Keywords:** Gestational diabetes mellitus, Mollicutes, *Ureaplasma urealyticum*, *Mycoplasma hominis*, Adverse pregnancy outcomes

## Abstract

**Background:**

The association of genital Mollicutes infection transition with adverse pregnancy outcomes was insignificant among general pregnant women, but there remains a paucity of evidence linking this relationship in gestational diabetes mellitus (GDM) women. The aim was to investigate the association between genital Mollicutes infection and transition and adverse pregnancy outcomes in GDM women, and to explore whether this association still exist when Mollicutes load varied.

**Methods:**

We involved pregnant women who attended antenatal care in Chongqing, China. After inclusion and exclusion criteria, we conducted a single-center cohort study of 432 GDM women with pregnancy outcomes from January 1, 2018 to December 31, 2021. The main outcome was adverse pregnancy outcomes, including premature rupture of membrane (PROM), fetal distress, macrosomia and others. The exposure was Mollicutes infection, including *Ureaplasma urealyticum* (Uu) and *Mycoplasma hominis* (Mh) collected in both the second and the third trimesters, and testing with polymerase chain reaction method. The logistic regression models were used to estimate the relationship between Mollicutes infection and adverse pregnancy outcomes.

**Results:**

Among 432 GDM women, 241 (55.79%) were infected with genital Mollicutes in either the second or third trimester of pregnancy. At the end of the pregnancy follow-up, 158 (36.57%) participants had adverse pregnancy outcomes, in which PROM, fetal distress and macrosomia were the most commonly observed adverse outcomes. Compared with the uninfected group, the Mollicutes (+/-) group showed no statistical significant increase in PROM (OR = 1.05, 95% CI:0.51 ∼ 2.08) and fetal distress (OR = 1.21, 95% CI: 0.31 ∼ 3.91). Among the 77 participants who were both Uu positive in the second and third trimesters, 38 participants presented a declined Uu load and 39 presented an increased Uu load. The Uu increased group had a 2.95 odds ratio (95% CI: 1.10~8.44) for adverse pregnancy outcomes.

**Conclusion:**

Mollicutes infection and transition during trimesters were not statistically associated with adverse pregnancy outcomes in GDM women. However, among those consistent infections, women with increasing Uu loads showed increased risks of adverse pregnancy outcomes. For GDM women with certain Mollicutes infection and colonization status, quantitative screening for vaginal infection at different weeks of pregnancy was recommended to provide personalized fertility treatment.

**Supplementary Information:**

The online version contains supplementary material available at 10.1186/s12884-024-06418-x.

## Introduction

Gestational diabetes mellitus (GDM), which is defined by the World Health Organization as glucose intolerance first recognized during pregnancy, has drawn global concern [1, 2]. The GDM incidence is alarming, affecting 14.8% of pregnant women in China [3] and presenting an increasing trend worldwide [4]. Researchers found that GDM was associated with a range of adverse pregnancy outcomes, such as preeclampsia, macrosomia, respiratory distress syndrome and so on [5]. Meanwhile, genital Mollicutes infection is a threat to reproductive health of females and the most common pathogens were *Ureaplasma urealyticum* (Uu) and *Mycoplasma hominis* (Mh). Previous studies found that genital Mollicutes infection was a risk factor for pregnant women and infants’ health [6, 7], and was related to sex hormone variation [8]. In practice, the association between hyperglycemia and the risk of infection has been well-established [9, 10]. Nevertheless, the association of Mollicutes infection and its load transition during trimesters on adverse pregnancy outcomes among GDM pregnant women has not been conclusively established.

Previous studies on genital infection and adverse pregnancy outcomes were conducted in observational studies of pregnant women with and without diabetic conditions during pregnancy [11–13], and no prospective cohort study has been conducted merely in the GDM population. Due to the specificity of genital Mollicutes in GDM patients and limited sample size, previous studies did not evaluate the infection of certain Mollicutes like Mh or Uu, nor did they explore the transition of Mollicutes from the second to the third trimester of pregnancy in GDM patients, nor did they explore Mollicutes load variation and adverse pregnancy outcomes. Considering the non-reassurance of embryo implantation, ectoplacental cavities and fetal status and low feasibility in the early trimester, vaginal swabs were conducted in the second and third trimester of pregnancy [14]. Therefore, we conducted a prospective, single-center cohort study to investigate whether genital Uu and Mh infection and their transition during trimesters were associated with adverse pregnancy outcomes in GDM patients, to assess the risk of adverse pregnancy outcomes with infection in both trimesters, and to further analyze the relationship between Mollicutes load variation and outcomes.

## Methods

### Study participants

**Fig. 1:**
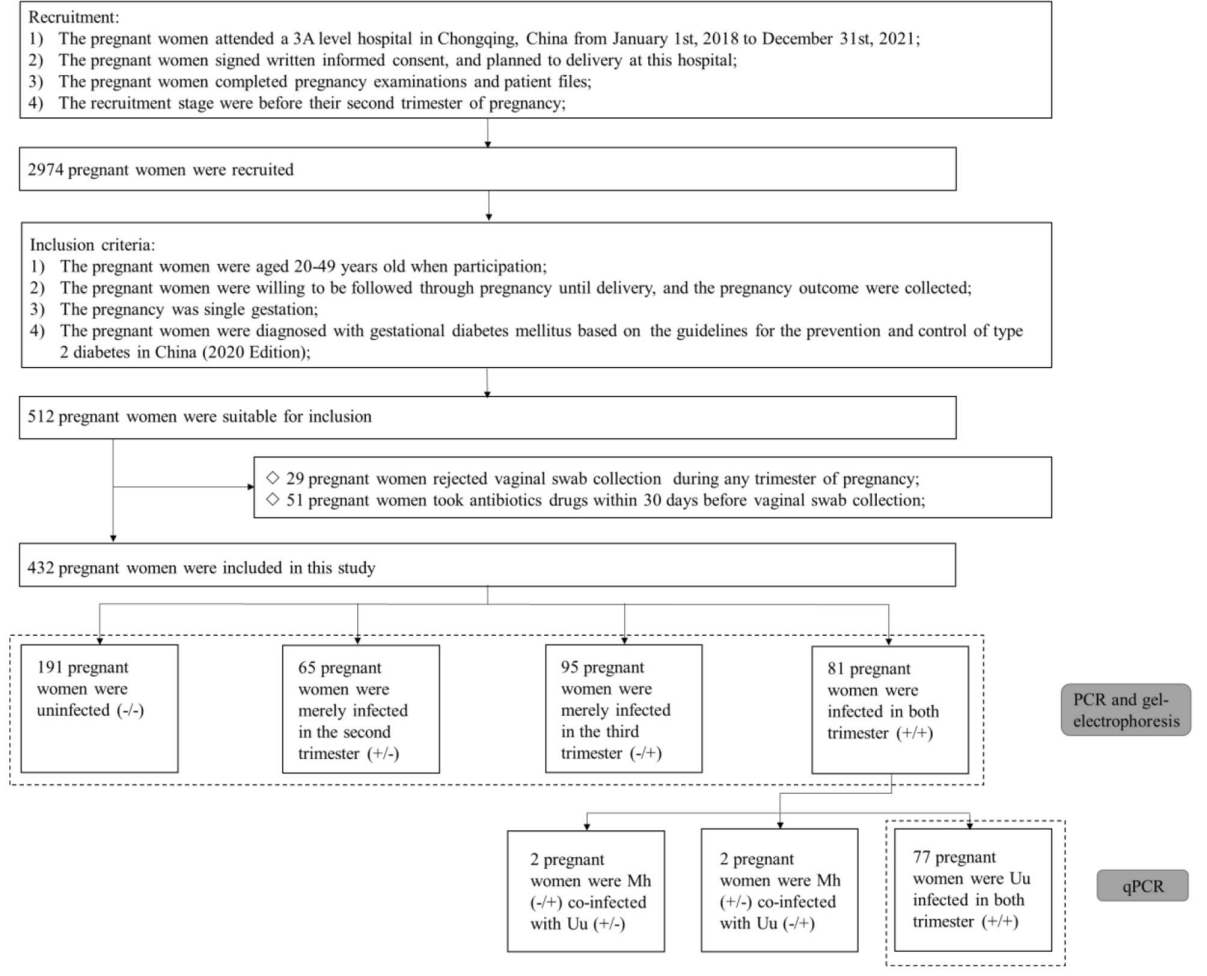
Flowchart for the study population

This prospective cohort study was reviewed and initiated by a 3 A level hospital in Chongqing, China to reduce the incidence of adverse pregnancy outcomes. We involved 2974 pregnant women planning to deliver at this hospital before their second trimester of pregnancy to enroll in our project from January 1, 2018 to December 31, 2021. After signing written informed consent, they completed pregnancy examinations and patient files and were followed up on delivery outcomes until pregnancy. All participants were involved before the second trimester of pregnancy. We included pregnant women who met the following criteria: (1) aged 20–49 years; (2) were willing to be followed until through pregnancy; (3) singleton gestation; (4) diagnosed with GDM, which followed the guidelines for the prevention and control of type 2 diabetes in China (2020 Edition) [15]. Women were excluded if they (1) rejected vaginal swab collection during any trimester of pregnancy; (2) taking antibiotics drugs within 30 days before vaginal swab collection (Fig. 1). Finally, 432 participants with GDM were included in this study. This study was authorized by the Institutional Ethics Committees of this hospital (Approval notice: 20,204,402), abided by the Declaration of Helsinki, and followed the Strengthening the Reporting of Observational Studies in Epidemiology (STROBE) reporting guideline.

### Study procedure

All participants were recruited and set up their initial prenatal files for the first time coming to the antenatal care center in the first trimester of pregnancy. Regular general and obstetric examinations were carried out every two to four weeks during trimesters. Pregnancy outcomes were observed and classified after delivery. Trained clinical physicians collected participants’ self-reported sociodemographic characteristics (including age and educational level) and menstrual/reproduction history (including pregnancy history and menstrual regularity) through standardized questionnaires through face-to-face interviews. Participants’ health status/lifestyles (including body mass index (BMI), alcohol intake, tobacco exposure, hypertension, cholesterol and hepatic functional markers) were obtained by questionnaires, physical examinations, or biochemical analyses. Participants would not be intervened or treated if merely colonized with Mollicutes based on current consensus on the diagnosis and treatment of mycoplasma by China Institute of Medical Professional Committee of the Reproductive Tract Infection Group [16].

Swabs were collected from all participants twice during the second and the third trimester (before 34 weeks of gestation) respectively at the lithotomy site. During the speculum examination, the gynecologist gently scrubbed the vaginal secretions with dry sterile cotton swabs from the posterior fornix by rotating them three times. Collected swabs were stored immediately upon collection in drying tubes with identification labels and transferred into a special collection container with a temperature of 4 °C. Furthermore, they were then placed in a -80 °C refrigerator until DNA extraction procedure was conducted. Drikold was utilized for sample transportation.

### Exposure measurements

DNA was isolated from vaginal swabs using Tiangen bacterial genome DNA extraction kit (TIANGEN, Beijing, China) and was used for template. Genital Mollicutes, including Mh and Uu, were assessed by polymerase chain reaction (PCR) amplification with 2 µL of template (extracted DNA), 1 µL of primer sets (Supplement Table 1), 12.5 µL of Taq polymerase and 8.5 µL of sterilized water. The PCR amplification protocol was as follows: 5 min at 95 °C for initial denaturation, then 40 cycles of 30 s at 95 °C for denaturing and 40 s at 58–60 °C for annealing and extension at 72 °C for 5 min. A 1% agarose gel-electrophoresis was performed to confirm the size of the PCR product. PCR and get-electrophoresis tests were performed within the second trimester and the third trimester of vaginal swabs of each participant.

After detecting a positive result of genital Mollicutes between the two pregnancy trimesters, quantitative PCRs (qPCRs) proceeded to figure out the quantitative load transition in genital tract from the second to the third trimester. The standard curve was made through the plasmid standard to quantified number of Uu. For the standard curve, a 10-fold serial dilution of the plasmid was prepared.

Each qPCR reaction system contained 20 µL, including 10 µL of SYBR qPCR Master Mix (Vazyme, Nanjing, China), 2 µL of DNA template, 0.4 µL of each primer, and 7.2 µL of sterile water. qPCR cyclic conditions started from initial denaturation at 94 °C for 30s, followed by 40 cycles of denaturation at 94 °C for 10 s, annealing at 58 °C for 30 s and extension. DNA templates were replaced with sterile water as no template controls. All samples were detected three times to ensure accuracy of the experimental results and to avoid errors caused by the operation. qPCR results were presented as threshold cycle (Ct) values. All participants were grouped based on the second and third trimester infection status as: uninfected (-/-), merely infected in the second trimester (+/-), merely infected in the third trimester (-/+), infected in both trimesters (+/+). Among those who were infected in both trimesters, Mollicutes load variation was calculated by ΔCt value, which was obtained by subtracting Ct in the third trimester from Ct in the second trimester for each participant. As Ct values were used as a proxy for viral load, with lower Ct values representing higher viral loads, ΔCt less than 0 was considered as reduced Mollicutes load from the second to the third trimester, and vice versa.

### Ascertainment of outcome

The main outcome was pregnancy outcomes, which included normal and adverse pregnancy outcomes. Adverse pregnancy outcomes included adverse maternal and fetal outcomes, containing premature rupture of membrane (PROM), fetal distress, macrosomia and others (oligohydramnios, polyhydramnios, placental abruption, chorioamnionitis, placenta accrete or percreta, amniotic fluid pollution, severe preeclampsia, postpartum haemorrhage, cholestasis of pregnancy, low birthweight, fetal growth restriction and birth defects). PROM was defined as rupture of membranes before the onset of labor [17]. Fetal distress was defined as any one of the following: meconium, fetal heart rate > 160, fetal heart rate < 120 bpm or abnormal electronic fetal heart rate monitoring [18]. Macrosomia was defined as birth weight ≥ 4000 g [19]. Oligohydramnios and polyhydramnios were defined as an amniotic fluid index of 5 cm or less and 24 cm or more [20], respectively. Placental abruption was defined as the premature separation of the implanted placenta before delivery [21]. Chorioamnionitis was defined as clinical suspicion of chorioamnionitis by obstetricians and later proven on placental histopathology [22]. A normal pregnancy outcome was defined as a full-term (delivery at or after the 37th week of pregnancy) live birth without the above mentioned abnormal outcomes [23].

### Covariates definition

This study selected covariates based on previous research on adverse pregnancy outcomes or genital infections. BMI was calculated by weight/height^2^ (kg/m^2^) and then categorized into 3 groups: underweight (< 18.5 kg/m^2^), normal weight (18.5–23.9 kg/m^2^), overweight and obese (≥ 24 kg/m^2^). We defined alcohol intake as drinking once a week, no matter how much alcohol is consumed. Tobacco exposure was defined as either active (smoking 1 cigarette per day for at least 1 year) or passive (exposure to environmental tobacco smoke on a daily basis). Hypertension referred to systolic blood pressure ≥ 140 mmHg and/or diastolic blood pressure ≥ 90 mmHg. Biochemical analyses were categorized based on the reference value in local laboratory. Clinical categories were combined if the maximum or minimum value was included in the reference value.

### Statistical analysis

For sample size calculation, the cohort study design was considered. A ratio of one uninfected woman for each infected woman was considered, with an adverse pregnancy occurrence of 0.16 in the uninfected and 0.46 in the infected group [24], which was estimated to achieve a confidence level of 95% and a power of 90%. Given an expected dropout rate of 10%, a minimum sample of 100 participants was estimated. Considering sufficient samples are essential in each subgroup, we enrolled as many participants as possible.

We described the baseline characteristics of the study population in terms of means (standard deviation) and counts (percentages). Student’s t-test, analysis of variance (ANOVA), Chi-square test and Fisher exact test were used to analyze the differences between groups. The logistic regression models were used to estimate odds ratios (ORs) and corresponding 95% confidence intervals (CIs). Adjustments were made among covariates which were different at baseline among groups. For the total participants, model A was adjusted for tobacco exposure and alcohol intake. Similarly, for participants infected in both trimesters, model A was adjusted for total cholesterol level. Analyses were conducted using R statistical software (version 4.1.3). Sample size was calculated using PASS software (version 15). A two-sided *p*-value < 0.05 was considered statistically significant.

## Results

Among these 432 female participants, 241 (55.79%) were infected with genital Mollicutes in either the second or third trimester of pregnancy. Among them, 142 (21.87%) were infected with Uu and 6 (1.38%) were infected with Mh during the second trimester, and the infection rates increased in the third trimester (39.12% and 2.78% for Uu and Mh, respectively). The proportion of participants with tobacco exposure and alcohol intake were statistically lower among participants who were merely infected in the third trimester. The baseline characteristics of the study population were presented in Table 1. Clinical biochemical analyses were shown in Table 2.

**Table 1:** Baseline characteristics of the study population

Variables	(-/-) No. (%)	(+/-) No. (%)	(-/+) No. (%)	(+/+) No. (%)	F/χ^2^	*P*
**All participants**	191	65	95	81		
**Sociodemographic characteristics**						
Age, years (mean ± SD)	31.55 ± 3.57	31.43 ± 3.56	31.60 ± 3.75	31.10 ± 3.23	0.51	0.47
20–34	154 (80.63)	56 (86.15)	74 (77.89)	64 (79.01)	1.85	0.60
35–49	37 (19.37)	9 (13.85)	21 (22.11)	17 (20.99)		
Missing	0	0	0	0		
Education level						
High school or below	28 (14.66)	14 (21.54)	12 (12.64)	12 (14.81)	-	0.24^a^
Bachelor degree	142 (74.35)	47 (72.31)	70 (73.68)	65 (80.25)		
Master degree or above	21 (10.99)	4 (6.15)	13 (13.68)	4 (4.94)		
Missing	0	0	0	0		
**Health status/lifestyles**						
BMI, kg/m^2^ (mean ± SD)	21.25 ± 3.51	21.54 ± 2.89	21.17 ± 2.68	20.94 ± 2.81	0.51	0.48
Underweight (< 18.50)	24 (12.57)	11 (16.92)	14 (14.74)	12 (14.81)	5.35	0.50
Normal (18.50–23.90)	144 (75.39)	40 (61.54)	65 (68.42)	57 (70.37)		
Overweight and obesity (≥ 24.00)	23 (12.04)	14 (21.54)	16 (16.84)	12 (14.82)		
Missing	0	0	0	0		
Hypertension						
Yes	1 (0.52)	1 (1.54)	2 (2.11)	3 (3.70)	-	0.19^a^
No	190 (99.48)	64 (98.46)	93 (97.89)	78 (96.30)		
Missing	0	0	0	0		
Tobacco exposure						
Yes	2 (1.06)	0 (0.00)	8 (8.51)	0 (0.00)	-	< 0.01^a^
No	187 (98.94)	63 (100.00)	86 (91.49)	81 (100.00)		
Missing	2	2	1	0		
Alcohol intake						
Yes	18 (9.42)	8 (12.31)	21 (22.11)	5 (6.17)	12.96	< 0.01
No	173 (90.58)	57 (87.69)	74 (77.89)	76 (93.83)		
Missing	0	0	0	0		
**Menstrual/reproduction history**						
Regular menstruation						
Yes	177 (92.67)	59 (90.77)	83 (87.37)	75 (92.59)	2.46	0.48
No	14 (7.33)	6 (9.23)	12 (12.63)	6 (7.41)		
Missing	0	0	0	0		
Number of gestation						
1	116 (60.73)	38 (58.46)	64 (67.37)	58 (71.60)	4.24	0.24
≥ 2	75 (39.27)	27 (41.54)	31 (32.63)	23 (28.40)		
Missing	0	0	0	0		
Number of parturition						
0	72 (37.70)	22 (33.85)	36 (37.89)	38 (46.91)	3.04	0.38
≥ 1	119 (62.30)	43 (66.15)	59 (62.11)	43 (53.09)		
Missing	0	0	0	0		

Note: ^a^ Fisher exact test were used. Data presented as n (%), unless noted otherwise. Participants were grouped based on the second and third trimester infection status as: uninfected in either trimester (-/-), merely infected in the second trimester (+/-), merely infected in the third trimester (-/+), infected in both trimesters (+/+). **Abbreviations**: BMI = body mass index; SD = standard deviation

**Table 2:** Clinical biochemical analyses of the study population

Variables	(-/-) No. (%)	(+/-) No. (%)	(-/+) No. (%)	(+/+) No. (%)	F/χ^2^	*P*
**All participants**	191	65	95	81		
**Blood lipids**						
Total cholesterol, mmol/L (mean ± SD)	5.04 ± 0.94	5.16 ± 1.01	5.17 ± 0.99	4.90 ± 0.81	0.25	0.62
0.00-5.20	123 (64.40)	43 (66.15)	56 (58.95)	57 (70.37)	2.58	0.46
≥ 5.21	68 (35.60)	22 (33.85)	39 (41.05)	24 (29.63)		
Missing	0	0	0	0		
Low density lipoprotein cholesterol, mmol/L (mean ± SD)	2.48 ± 0.70	2.58 ± 0.66	2.53 ± 0.73	2.35 ± 0.62	0.96	0.33
0.00-3.10	158 (82.72)	52 (80.00)	73 (76.84)	71 (87.65)	3.70	0.30
≥ 3.11	33 (17.28)	13 (20.00)	22 (23.16)	10 (12.35)		
Missing	0	0	0	0		
High density lipoprotein cholesterol, mmol/L (mean ± SD)	1.85 ± 0.34	1.78 ± 0.37	1.87 ± 0.37	1.86 ± 0.34	0.11	0.74
0.00-1.80	86 (45.50)	35 (53.85)	40 (42.11)	41 (51.25)	2.89	0.41
≥ 1.81	103 (54.50)	30 (46.15)	55 (57.89)	39 (48.75)		
Missing	2	0	0	1		
Triglyceride, mmol/L (mean ± SD)	1.60 ± 0.77	1.93 ± 1.01	1.70 ± 0.75	1.56 ± 0.66	0.01	0.95
0.00-1.70	128 (67.02)	34 (52.31)	60 (63.16)	59 (72.84)	7.28	0.06
≥ 1.71	63 (32.98)	31 (47.69)	35 (36.84)	22 (27.16)		
Missing	0	0	0	0		
**Nutrition/immune indicators**						
Albumin, g/L (mean ± SD)	41.96 ± 3.43	41.83 ± 3.53	41.97 ± 3.78	42.38 ± 3.67	21.73	0.24
< 40.00	44 (23.04)	16 (24.62)	25 (26.32)	16 (19.75)	1.12	0.77
40.00–55.00	147 (76.96)	49 (75.38)	70 (73.68)	65 (80.25)		
Missing	0	0	0	0		
Globulin, g/L (mean ± SD)	26.81 ± 3.53	27.23 ± 4.05	27.19 ± 3.78	27.44 ± 4.06	0.55	0.46
< 20.00	3 (1.57)	0 (0.00)	1 (1.05)	2 (2.47)	-	0.75^a^
20.00–40.00	188 (98.43)	65 (100.00)	94 (98.95)	79 (97.53)		
Missing	0	0	0	0		
**Hepatic function indexes**						
Alanine aminotransferase, U/L (Median, Quantile)	17.99 ± 12.65	18.71 ± 13.92	20.11 ± 17.11	21.62 ± 24.59	3.02	0.08
< 7.00	13 (6.81)	5 (7.69)	3 (3.16)	3 (3.70)	-	0.66^a^
7.00–40.00	166 (86.91)	56 (86.15)	83 (87.37)	70 (86.42)		
≥ 40.01	12 (6.28)	4 (6.15)	9 (9.47)	8 (9.88)		
Missing	0	0	0	0		
Aspartate aminotransferase, U/L (mean ± SD)	19.70 ± 6.81	19.80 ± 6.55	20.24 ± 8.23	20.91 ± 12.38	1.19	0.28
< 13.00	17 (8.90)	5 (7.69)	10 (10.53)	3 (3.70)		0.36^a^
13.00–35.00	170 (89.01)	59 (90.77)	80 (84.21)	74 (91.36)		
≥ 35.01	4 (2.09)	1 (1.54)	5 (5.26)	4 (4.94)		
Missing	0	0	0	0		

Note: ^a^ Fisher exact test were used. Data presented as n (%), unless noted otherwise. Participants were grouped based on the second and third trimester infection status as: uninfected in either trimester (-/-), merely infected in the second trimester (+/-), merely infected in the third trimester (-/+), infected in both trimesters (+/+). **Abbreviations**: SD = standard deviation

At the end of the pregnancy follow-up period, 158 (36.57%) GDM participants had adverse pregnancy outcomes. When further stratified by Mollicutes clade, participants with Uu infection in both trimesters presented slightly lower adverse pregnancy outcome rates ((-/-), (+/-), (-/+), (+/+): 37.37%, 35.38%, 36.96% and 35.06% respectively), while participants with Mh infection presented a wide range ((-/-), (+/-), (-/+), (+/+): 37.50%, 25.00%, 10.00% and 0.00% respectively) because of the low prevalence rate of Mh in pregnant women in China. However, there was no statistical significance between the four infection statuses (Table 3).

**Table 3:** Association of different genital Mollicutes infection statuses in the second and the third trimester with adverse pregnancy outcomes

Groups	Normal pregnancy outcomes No. (%)	Adverse pregnancy outcomes No. (%)	χ^2^	*P*
**Genital Mollicutes infection**				
(-/-)	117 (61.26)	74 (38.74)	0.82	0.84
(+/-)	42 (64.62)	23 (35.38)		
(-/+)	61 (64.21)	34 (35.79)		
(+/+)	54 (66.67)	27 (33.33)		
***Ureaplasma urealyticum*** **infection**				
(-/-)	124 (62.63)	74 (37.37)	0.18	0.98
(+/-)	42 (64.62)	23 (35.38)		
(-/+)	58 (63.04)	34 (36.96)		
(+/+)	50 (64.94)	27 (35.06)		
***Mycoplasma hominis*** **infection**				
(-/-)	260 (62.50)	156 (37.50)	-	0.21^a^
(+/-)	3 (75.00)	1 (25.00)		
(-/+)	9 (90.00)	1 (10.00)		
(+/+)	2 (100.00)	0 (0.00)		

^a^ Fisher exact test were used. Participants were grouped based on the second and third trimester infection status as: uninfected in either trimester (-/-), merely infected in the second trimester (+/-), merely infected in the third trimester (-/+), infected in both trimesters (+/+)

Among those in all pregnancy outcomes, PROM was the most commonly observed adverse pregnancy outcome (22.69%), which was also true when grouped by infection status and period, regardless of whether participants were uninfected (22.51%), merely infected in the second trimester (21.54%), merely infected in the third trimester (22.11%) or infected in both trimesters (24.69%). The second and third rank of adverse pregnancy outcomes were fetal distress (4.63%) and macrosomia (3.01%). Compared with the uninfected group, the Mollicutes (+/-) group showed a slightly increased odds ratio in PROM and fetal distress after adjustment (Table 4). All infection statuses had lower ORs in macrosomia and other adverse pregnancy outcomes (Table 4). This was also true after adjustment. However, no statistical significance was observed due to the small sample size after grouping by infection status from a low Mollicutes infection rate.

**Table 4:** Association of different genital Mollicutes infection statuses in the second and the third trimester with premature rupture of membrane, fetal distress macrosomia and other adverse pregnancy outcomes

Outcomes	*N*	No. of events	%	Crude Model		Model A	
				OR	95% CI	OR	95% CI
**Premature rupture of membrane**							
(-/-)	191	43	22.51	1.00		1.00	
(+/-)	65	14	21.54	0.94	(0.47, 1.83)	1.05	(0.51, 2.08)
(-/+)	95	21	22.11	0.98	(0.53, 1.75)	0.87	(0.46, 1.59)
(+/+)	81	20	24.69	1.13	(0.61, 2.06)	0.99	(0.53, 1.82)
**Fetal distress**							
(-/-)	191	10	5.24	1.00		1.00	
(+/-)	65	4	6.15	1.19	(0.32, 3.69)	1.21	(0.31, 3.91)
(-/+)	95	4	4.21	0.80	(0.21, 2.45)	0.69	(0.18, 2.19)
(+/+)	81	2	2.47	0.46	(0.07, 1.79)	0.40	(0.06, 1.58)
**Macrosomia**							
(-/-)	191	8	4.19	1.00		1.00	
(+/-)	65	2	3.08	0.73	(0.11, 2.99)	0.72	(0.11, 2.98)
(-/+)	95	2	2.11	0.49	(0.07, 2.01)	0.55	(0.08, 2.26)
(+/+)	81	1	1.23	0.29	(0.02, 1.60)	0.29	(0.02, 1.65)
**Others**							
(-/-)	191	25	13.09	1.00		1.00	
(+/-)	65	3	4.62	0.32	(0.07, 0.96)	0.33	(0.08, 1.00)
(-/+)	95	10	10.53	0.78	(0.34, 1.66)	0.68	(0.29, 1.48)
(+/+)	81	5	6.17	0.44	(0.14, 1.10)	0.41	(0.13, 1.04)

*N* is the total number in this category. No. of events is the number in the category with the outcome, and % is the proportion in the category with the outcome. Participants were grouped based on the second and third trimester infection status as: uninfected in either trimester (-/-), merely infected in the second trimester (+/-), merely infected in the third trimester (-/+), infected in both trimesters (+/+). Model A was adjusted for tobacco exposure and alcohol intake

We further performed quantification Uu load detection among the 77 participants who were both positive in the second and third trimesters. Because of the low prevalence rate of Mh, we did not consider the situation of Mh infection, including 2 participants with Uu (-/+) coinfected with Mh (+/-) and 2 participants with Uu (+/-) coinfected with Mh (-/+). Based on Uu load detection, the mean (SD) Ct values in the second and third trimesters were 27.04 (3.61) and 27.02 (3.27), respectively. Among them, 38 participants presented a declined Uu load (Uu declined group) and 39 presented an increased Uu load (Uu increased group) from the second to third pregnancy trimester. The baseline characteristics and biochemical analyses were shown in Supplement Tables 2 and 3. The incidence of adverse pregnancy outcomes was 35.06% in Uu (+/+) patients with GDM. The participants in the Uu declined group had an incidence of 23.69% adverse pregnancy outcome, which was statistically lower than that in participants in Uu increased group (46.16%) (Table 5). Uu increased group had a 2.95 odds ratio (95% CI: 1.10 ∼ 8.44) for adverse pregnancy outcomes compared with Uu declined group (Table 5). The top four frequent adverse events were PROM, fetal distress, macrosomia and chorioamnionitis, which was similar to observation among the total participants.

**Table 5:** Association of genital *Ureaplasma urealyticum* load change with adverse pregnancy outcomes according to women infected in both trimesters

Groups	*N*	No. of events	%	Crude Model		Model A	
				OR	95% CI	OR	95% CI
**Uu load declined**	38	9	23.69	1.00		1.00	
**Uu load increased**	39	18	46.16	2.76	(1.06,7.60)	2.95	(1.10,8.44)

N is the total number in this category. No. of events is the number in the category with the outcome, and % is the proportion in the category with the outcome. Model A was adjusted for total cholesterol level

## Discussion

There are nearly one-fifth of pregnant women are diagnosed with GDM in mainland China [25], which is significantly higher than global average estimates [26], providing the feasibility of population basis for this study. With the resurgence of mycoplasma infection, especially in infants and children this autumn, continuous surveillance has been again put on the agenda [27]. Based on our pregnancy cohort, we found that genital Mollicutes varied in different stages of GDM pregnancies, which was similar to previous study [28]. Moreover, we explored that one third of GDM patients had adverse pregnancy outcomes, and it was pronounced in patients with increased Uu load among those with consistent Uu colonization, thereby filling some gaps in the field.

Previous population studies on the association between genital tract infection and adverse pregnancy outcomes have shown inconsistent results. Dalia et al. found that genital bacterial infections were significantly associated with PROM in GDM patients in India [29]. Observational studies found that abnormal flora was associated with PROM, puerperal infection and chorioamnionitis [30] among women with GDM [31]. Xiao et al. additionally found increased postpartum hemorrhage and fetal distress [32]. However, a meta-analysis suggested no association between genital Mollicutes infection and PROM or abnormal birth weight [33]. It is worth mentioning that prior relevant studies have mainly focused on the association between women with genital tract infection and adverse pregnancy outcomes, and few studies have focused on the exposure of Mollicutes infection leading to adverse pregnancy outcomes, let alone in the subgroup of the large GDM population. These alternative results were similar to ours.

Limited previous studies based on GDM patients only focused on transition of regular genital bacterial infections, ignoring the persistent colonization of ureaplasma, which was a non-bacterial and non-fungal microbiome, and its adverse effects. Therefore, studies assessing the correlation between Mollicutes transition and adverse pregnancy outcomes in GDM populations are rare. A multi-central cohort study on 127 GDM women found that Uu and Mh presented higher prevalence rates after the 28th gestational week [28]. Dalia et al. found that bacterial infection varied from the second to the third trimester, and infection at any time was positively related to PROM in pregnant women with GDM [29]. With 1.4 billion people, China has a highly diverse vaginal environment compared to other ethnic groups, providing a wide range of possibilities to assess the load transition among women with consistent Mollicutes infection and colonization. We unexpectedly found that the association between Uu infection and adverse pregnancy outcomes was more pronounced in women with increased Uu load than with decreased Uu load in a longer infection and colonization period.

There is no clear mechanism explanation for how Mollicutes infection transition in a longer colonization period results in adverse pregnancy outcomes [34]. Hypotheses were focused on the long term effects of epithelial cell damage and chronic inflammation. For PROM and chorioamnionitis, some findings suggested that ureaplasma infection was associated with cervical epithelial cell damage [35], leading to a more substantial possibility of increased ascending infection. After cervical damage occurred, there were significant increases in both ureaplasma titres increase and ascending ureaplasma infection [36], which appeared to be correlated with PROM [37, 38] and chorioamnionitis [39]. Notably, these infections may also trigger chronic inflammation and affect local and cellular immunity in the cervix. Ioannis [36] found that ureaplasma infection stimulates the production of proinflammatory cytokines, reflected by the significant positive relationship of levels of cytokine expression and ureaplasma derived gene ureC through TLR signalling, especially increased TNFα, IL-1β, CXCL-1 and CXCL-2 cytokine expression in foetal membranes, placenta and the myometrium. In in vitro studies, ureaplasma had similar upregulate effects in TNFα, IL-1β, IL-6 and IL-8 [40–42], which were commonly associated with PROM or chorioamnionitis [43]. It may be speculated that induction of PROM and chorioamnionitis might be a consequence of an infection-mediated inflammatory response via the increase of ureaplasma load during pregnancy, promoting the release of several inflammatory factors. For fetal distress and macrosomia, the hypotheses about the mechanism were mainly focused on the possible indirect effect of infection on blood glucose elevation. Infection status would substantially alter the norm of blood glucose dynamics despite injecting insulin and reducing carbohydrate consumption [44]. The increase of infection related indexes, for example, serum levels of IL-1β, TNF-α, and IL-6, were significantly associated with a blood glucose rise [45]. By exacerbating inflammation and further increasing hyperglycemia, which was strongly associated with macrosomia [46] and fetal distress [47], the increase of ureaplasma load showed a greater effect in adverse pregnancy outcomes. However, the direct mechanism of Uu load transition, GDM and adverse pregnancy outcomes still remain unclear.

The present study has several strengths. First, in contrast to previous studies that focus on populations comparing healthy and GDM pregnant women, we for the first time focused on the high risk group to further investigate the association between genital Mollicutes infection and adverse pregnancy outcomes in a prospective cohort of GDM pregnancy women, embodied the population of such studies. Second, we assessed for the first time the adverse pregnancy outcome of GDM women with infection transition during trimesters. Third, we performed Mollicutes load based on Uu and found that the association between Uu infection and adverse pregnancy outcomes was more pronounced in GDM pregnant women with increased Uu load.

### Limitations

Our first limitation was human, material, and financial constraints which prevented us from collecting more detailed data on Mollicutes infection status, such as the exact time of Mollicutes infection, multiple genital infection tests, complications or treatment information, which limited further in-depth analysis of GDM with different infection status. Second, we merely detected common Mollicutes in female genital tract, neglecting the infection of *Mycoplasma Genitalium* because of its low prevalence rate. Third, since the blood glucose level was not monitored during the whole pregnancy trimester and GDM status was transformed into categorical dichotomous data, we were unable to perform further subgroup analysis such as classified glucose levels and GDM severity. Fourth, it is inevitable that there would be bias in this study due to the self-reported information on behavioral characteristics and menstruation. Fifth, because vaginal swabs were not collected during the first trimester of pregnancy because of the non-reassuring of fetal status, Mollicutes infection transition surveillance could not start from the early stage of pregnancy. Sixth, since we studied only Chinese GDM patients in a single center which is larger than previous single-center studies, it is important to take caution when extrapolating the results to other ethnicities.

## Conclusion

Based on this pregnancy cohort study, we found no statistically significant association between Molicutes infection and transition and adverse pregnancy outcomes in GDM patients. However, when assessing for the first time the relationship between ureaplasma load variation and adverse pregnancy outcomes, we found that among GDM women with infection in both trimesters, the risk of adverse pregnancy outcomes was significantly increased in GDM pregnant women with increased ureaplasma load from the second to the third trimester. This study highlights the need for quantitative screening for vaginal infections at different weeks of pregnancy in persistently infected GDM women and for careful personalized fertility treatment during perinatal period.

### Electronic supplementary material

Below is the link to the electronic supplementary material.

Supplementary Material 1

## Data Availability

The datasets used and/or analysed during the current study are available from the corresponding author Bei Wang (first corresponding author) or Junhui Zhang on reasonable request.

## Abbreviations

BMI: Body mass index
CI: Confidence interval
GDM: Gestational diabetes mellitus, Mh, *Mycoplasma hominis*
OR: Odds ratio
PROM: Premature rupture of membrane
UU: *Ureaplasma urealyticum*
