# Supplementary material for: Transition of the genital mollicutes from the second to the third trimester of pregnancy and its association with adverse pregnancy outcomes in GDM women: a prospective, single-center cohort study from China
Source: BMC Pregnancy Childbirth. 2024 Apr 3;24:233. doi: 10.1186/s12884-024-06418-x (PMC10993520; doi:10.1186/s12884-024-06418-x)
Supplement: Supplementary file 1 — Supplementary Material 1 [file 12884_2024_6418_MOESM1_ESM.docx]

**Supplement Table 1 PCR primers for PCR and qPCR assay**

| **Target species** | **Primer sequencing** | **Amplicon (bp)** | **Tm (°C)** |
| --- | --- | --- | --- |
| ***Ureaplasma urealyticum*** | F: 5’-CAATCTGCTCGTGAAGTATTAC-3’  R: 5’-ACGACGTCCATAAGCAACT-3’ | 429 | 58 |
| ***Mycoplasma hominis*** | F: 5’-CAATGGCTAATGCCGGATACGC-3’  R: 5’-GGTACCGTCAGTCTGCAAT-3’ | 331 | 60 |

**Supplement Table 2 Baseline characteristics of Uu load increased group and Uu load declined group in participants infected in both second and third trimesters**

| **Variables** | | **Uu load declined**  **No. (%)** | **Uu load increased**  **No. (%)** | **t/*χ*^2^** | ***P*** |
| --- | --- | --- | --- | --- | --- |
| **All participants** | | 38 | 39 |  |  |
| **Sociodemographic characteristics** | |  |  |  |  |
|  | Age, years (mean ± SD) | 31.24±3.65 | 30.82±2.81 | 0.56 | 0.58 |
|  | 20-34 | 28 (73.68) | 33 (84.62) | 0.81 | 0.37 |
|  | 35-49 | 10 (26.32) | 6 (15.38) |  |  |
|  | Missing | 0 | 0 |  |  |
|  | Education level |  |  |  |  |
|  | High school or below | 6 (15.79) | 6 (15.38) | - | 1.00^a^ |
|  | Bachelor degree | 30 (78.95) | 31 (79.49) |  |  |
|  | Master degree or above | 2 (5.26) | 2 (5.13) |  |  |
|  | Missing | 0 | 0 |  |  |
| **Health status/lifestyles** | |  |  |  |  |
|  | BMI, kg/m^2^ (mean ± SD) | 20.57±3.04 | 21.22±2.69 | -1.00 | 0.32 |
|  | Underweight (<18.50) | 7 (18.42) | 5 (12.82) | 1.13 | 0.57 |
|  | Normal (18.50-23.90) | 24 (63.16) | 29 (74.36) |  |  |
|  | Overweight and obesity (≥24.00) | 7 (18.42) | 5 (12.82) |  |  |
|  | Missing | 0 | 0 |  |  |
|  | Hypertension |  |  |  |  |
|  | Yes | 1 (2.63) | 2 (5.13) | - | 1.00^a^ |
|  | No | 37 (97.37) | 37 (94.87) |  |  |
|  | Missing | 0 | 0 |  |  |
|  | Tobacco exposure |  |  |  |  |
|  | Yes | 0 (0.00) | 0 (0.00) | - | 1.00^a^ |
|  | No | 38 (100.00) | 39 (100.00) |  |  |
|  | Missing | 0 | 0 |  |  |
|  | Alcohol intake |  |  |  |  |
|  | Yes | 3 (7.89) | 2 (5.13) | - | 0.67^a^ |
|  | No | 35 (92.11) | 37 (94.87) |  |  |
|  | Missing | 0 | 0 |  |  |
| **Menstrual/reproduction history** | |  |  |  |  |
|  | Regular menstruation |  |  |  |  |
|  | Yes | 34 (89.47) | 37 (94.87) | - | 0.43^a^ |
|  | No | 4 (10.53) | 2 (5.13) |  |  |
|  | Missing | 0 | 0 |  |  |
|  | Number of gestation |  |  |  |  |
|  | 1 | 25 (65.79) | 31 (79.49) | 1.20 | 0.27 |
|  | ≥2 | 13 (34.21) | 8 (20.51) |  |  |
|  | Missing | 0 | 0 |  |  |
|  | Number of parturition |  |  |  |  |
|  | 0 | 19 (50.00) | 17 (43.59) | 0.11 | 0.74 |
|  | ≥1 | 19 (50.00) | 22 (56.41) |  |  |
|  | Missing | 0 | 0 |  |  |

Note: ^a^ Fisher exact test were used. Data presented as n (%), unless noted otherwise. **Abbreviations:** BMI = body mass index; SD = standard deviation.

**Supplement Table 3 Clinical biochemical analyses of Uu load increased group and Uu load declined group in participants infected in both second and third trimesters.**

| **Variables** | | **Uu load declined**  **No. (%)** | **Uu load increased**  **No. (%)** | **t/*χ*^2^** | ***P*** |
| --- | --- | --- | --- | --- | --- |
| **All participants** | | 38 | 39 |  |  |
| **Clinical biomedical analyses** | |  |  |  |  |
| **Blood lipids** | |  |  |  |  |
|  | Total cholesterol, mmol/L (mean ± SD) | 5.09±0.83 | 4.76±0.79 | 1.75 | 0.08 |
|  | 0.00-5.20 | 21 (55.26) | 32 (82.05) | 5.25 | 0.02 |
|  | ≥5.21 | 17 (44.74) | 7 (17.95) |  |  |
|  | Missing | 0 | 0 |  |  |
|  | Low density lipoprotein cholesterol, mmol/L (mean ± SD) | 2.49±0.59 | 2.22±0.66 | 1.92 | 0.06 |
|  | 0.00-3.10 | 31 (81.58) | 36 (92.31) | - | 0.19^a^ |
|  | ≥3.11 | 7 (18.42) | 3 (7.69) |  |  |
|  | Missing | 0 | 0 |  |  |
|  | High density lipoprotein cholesterol, mmol/L (mean ± SD) | 1.90±0.37 | 1.85±0.30 | 0.84 | 0.36 |
|  | 0.00-1.80 | 16 (42.11) | 21 (55.26) |  |  |
|  | ≥1.81 | 22 (57.89) | 17 (44.74) |  |  |
|  | Missing | 0 | 1 |  |  |
|  | Triglyceride, mmol/L (mean ± SD) | 1.57±0.65 | 1.58±0.70 | -0.08 | 0.93 |
|  | 0.00-1.70 | 29 (76.32) | 27 (71.05) | 0.20 | 0.66 |
|  | ≥1.71 | 9 (23.68) | 12 (28.95) |  |  |
|  | Missing | 0 | 0 |  |  |
| **Nutrition/immune indicators** | |  |  |  |  |
|  | Albumin, g/L (mean ± SD) | 42.21±3.47 | 42.61±3.93 | -0.47 | 0.64 |
|  | <40.00 | 8 (21.05) | 6 (15.38) | 0.12 | 0.73 |
|  | 40.00-55.00 | 30 (78.95) | 33 (84.62) |  |  |
|  | Missing | 0 | 0 |  |  |
|  | Globulin, g/L (mean ± SD) | 26.84±3.77 | 27.88±4.44 | -1.11 | 0.27 |
|  | <20.00 | 1 (2.63) | 1 (2.56) | - | 1.00^a^ |
|  | 20.00-40.00 | 37 (97.37) | 38 (97.44) |  |  |
|  | Missing | 0 | 0 |  |  |
| **Hepatic function indexes** | |  |  |  |  |
|  | Alanine aminotransferase, U/L (Median, Quantile) | 18.74±15.57 | 24.74±30.79 | -1.06 | 0.30 |
|  | <7.00 | 2 (5.26) | 1 (2.56) | - | 0.89^a^ |
|  | 7.00-40.00 | 32 (84.21) | 34 (87.18) |  |  |
|  | ≥40.01 | 4 (10.53) | 4 (10.26) |  |  |
|  | Missing | 0 | 0 |  |  |
|  | Aspartate aminotransferase, U/L (mean ± SD) | 20.45±10.57 | 21.77±14.50 | -0.46 | 0.65 |
|  | <13.00 | 2 (5.26) | 1 (2.56) | - | 0.85^a^ |
|  | 13.00-35.00 | 34 (89.47) | 36 (92.31) |  |  |
|  | ≥35.01 | 2 (5.27) | 2 (5.13) |  |  |
|  | Missing | 0 | 0 |  |  |

Note: ^a^ Fisher exact test were used. Data presented as n (%), unless noted otherwise. **Abbreviations:** SD = standard deviation.
